# Rationale and Logistics of Continuous Infusion Cephalosporin Antibiotics

**DOI:** 10.3390/pharmacy12060185

**Published:** 2024-12-05

**Authors:** Abbie L. Blunier, R. Jake Crocker, Rachel Foster, Stephanie S. May, Caroline E. Powers, P. Brandon Bookstaver

**Affiliations:** 1 Department of Pharmacy, Mayo Clinic, 200 First Street SW, Rochester, MN 55905, USA; blunier.abbie@mayo.edu; 2 Department of Pharmacy, Prisma Health Upstate, 701 Grove Rd, Greenville, SC 29605, USA; ronald.crocker@prismahealth.org; 3 Department of Pharmacy, Intermountain Medical Center, 5121 South Cottonwood St, Murray, UT 84107, USA; rachel.foster@imail.org (R.F.); stephanie.may@imail.org (S.S.M.); 4 Infectious Diseases Telehealth Service, Intermountain Health, 5171 South Cottonwood St, Murray, UT 84107, USA; 5 Department of Pharmacy, Ralph H. Johnson VA Medical Center, 109 Bee St, Charleston, SC 29401, USA; caroline.powers@va.gov; 6 Department of Pharmacy, Prisma Health Richland, 5 Medical Park Drive, Columbia, SC 29203, USA; 7 Department of Clinical Pharmacy and Outcomes Sciences, University of South Carolina College of Pharmacy, 715 Sumter Street, Columbia, SC 29208, USA

**Keywords:** cephalosporins, beta-lactam antibiotics, continuous infusion, pharmacokinetics, pharmacodynamics, antimicrobial stewardship

## Abstract

Cephalosporins have traditionally been administered as an intermittent infusion. With the knowledge that cephalosporins demonstrate a time-dependent pharmacodynamic profile, administration via continuous infusion may provide more effective antibiotic exposure for successful therapy. Proposed benefits of administration via continuous infusion include less IV manipulation, decreased potential for antibiotic resistance, and potential cost savings. The objective of this review was to provide a detailed assessment of available evidence for the use of continuous infusion cephalosporins and practical dosing and administration recommendations. Studies were gathered and assessed for inclusion via a literature search of PubMed and Ovid MEDLINE using mesh terms [“continuous infusion” and “cephalosporin”], “intermittent infusion”, [“intermittent versus continuous” and “cephalosporin”], “continuous infusion cephalosporin”, as well as specific drug names. References from included studies were also evaluated for inclusion. Data which compared the two administration methods (continuous infusion vs. intermittent infusion) were evaluated. Thirty-five studies were analyzed among several cephalosporins with variable delivery. Dosing regimens utilized in the selected studies were assessed with known compatibility and stability data and further summarized.

## 1. Introduction

Since penicillin antibiotics were introduced in the 1940’s, there has been a growth of antimicrobial resistance and a need for both new active antibacterial treatments and to steward current treatment options. Cephalosporin antibiotics are generally considered broad-spectrum with activity against both gram-positive and gram-negative bacteria. One practical way to steward the use of currently available antimicrobial agents is to optimize pharmacokinetics and pharmacodynamics (PK/PD), specifically the probability of target attainment (PTA) [1]. Cephalosporins exhibit time-dependent bactericidal activity, highlighting the importance of maintaining levels above the minimum inhibitory concentration over time (fT > MIC) [2]. Parenteral cephalosporins are traditionally administered as intermittent infusions (IIs), often 0.5 to 1 h infusions every 8 to 12 h in the absence of impaired renal function, which may result in suboptimal concentrations in some situations. This is especially true in managing gram-negative bacteria, possibly leading to decreased antibiotic effectiveness and the potential risk for the development of antibiotic resistance. In contrast, administration of cephalosporins via extended (EI) or continuous infusion (CI) optimizes the drug’s fT > MIC, increasing the PTA [2,3]. Several beta-lactams, including cephalosporins, approved by the U.S. Food and Drug Administration (FDA) in recent years have manufactured labeled dosing that includes prolonged infusion times (e.g., 2–3 h). Use of CI as a delivery modality may further optimize the PK/PD, ease of administration, and potentially improve clinical outcomes in patients treated with cephalosporin antibiotics. A recent systematic review and meta-analysis demonstrated lower 90-day mortality in patients with sepsis or septic shock among those receiving prolonged infusions compared to IIs [4].

While administering cephalosporins as a CI will primarily impact PTA against gram-negative organisms, the practical benefits of CI for any patient may help alleviate some logistical concerns. These prolonged infusions of up to 24 h will lead to fewer intravenous (IV) site manipulations and, subsequently, a lower risk of IV-site and bloodstream infections. Subsequently, fewer healthcare workers or patient-facilitated IV bag changes will be necessary when administered in the outpatient setting. Leveraging CI has the potential for decreasing the required amount of drug and increasing patient mobilization. These advantages contribute to lower costs for the patient and the health care system while increasing patient quality of life.

One potential disadvantage of administration via CI in the outpatient setting is that it requires the patient to be attached to an IV infusion delivery mechanism (e.g., portable pump) for up to 24 h per day. Though this may not be ideal for some patients, II in the outpatient setting often encompasses three to four infusions per day. Multiple interruptions in the patient’s day for II may lead to lower patient satisfaction. Regimens requiring multiple infusions per day (e.g., 2–3) may also limit placement at hospital discharge for patients who are not able to return home for outpatient antibiotics.

Many studies of CI cephalosporins have been conducted describing their safety and effectiveness. The published evidence has suggested varying dosing, stability, and compatibility recommendations. There are limited resources to guide practical dosing and administration of CI cephalosporins; however, a recent consensus statement published by Hong et al. in 2023 provides recommendations from an expert international panel for the use of prolonged infusion beta-lactams including PD targets, therapeutic drug monitoring (TDM), and dosing recommendations [5]. A recent randomized control trial evaluated CI versus II beta-lactam antibiotics for reduction in all-cause mortality in critically ill patients with sepsis. While there was no difference found in the primary outcome, higher rates of clinical cure were observed in the CI group up to 14 days after randomization, which may suggest a potential benefit for early initiation of CIs for patients with sepsis [6]. This review herein will provide a detailed assessment of the available published evidence for CI cephalosporins against both gram-positive and gram-negative organisms. Practical dosing recommendations will be proposed for the most frequently used, FDA-approved parenteral cephalosporins to assist clinicians in the development of local dosing guidelines.

## 2. Materials and Methods

### 2.1. Literature Search

A literature search was performed via PubMed and Ovid MEDLINE from inception to May 2024 to gather articles assessing the efficacy of continuous infusion cephalosporins using mesh terms such as “continuous infusion”, “cephalosporin”, [“continuous infusion” and “cephalosporin”], “intermittent infusion”, [“intermittent versus continuous”], and “continuous infusion cephalosporin”, as well as specific drug names.

### 2.2. Study Selection

All articles published from 1980 to present (May 2024) that utilized infusions of cephalosporin antibiotics were reviewed including observational studies, controlled studies, and simulations. Four investigators reviewed the available literature for inclusion. References from these articles were also assessed for inclusion. Data are summarized below for each included agent. Data of purely confirmatory nature may have been excluded from cephalosporin summaries if investigators determined no new conclusions were gleaned. Table 1 and Table 2 contain dosing recommendations and compiled summary of the available evidence, respectively.

## 3. Results

### 3.1. Cephalosporins

#### 3.1.1. Cefazolin

Cefazolin (CFZ) is a first-generation cephalosporin most frequently used to treat gram-positive bacteria including methicillin-susceptible *Staphylococcus aureus* and *Streptococci* [3]. CFZ is used in the management of a variety of infections including acute bacterial skin and skin structure infections (ABSSSIs), bone and joint infections, bacteremia including endovascular infections, and surgical prophylaxis [86]. Like most other cephalosporins, CFZ is primarily renally excreted (65%) and is 80% protein bound. CFZ is traditionally dosed via 30 min infusions of 1–2 g IV every 8 h, with a maximum daily dose of 12 g per day [3]. Higher total daily dosing or modified dosing strategies may be needed for obese patients (>120 kg) and deep-seated infections [87].

CI CFZ has demonstrated safety and effectiveness in several populations, including patients receiving perioperative prophylaxis and outpatient IV antibiotic therapy for uncomplicated cellulitis, bone and joint infections, and central nervous system (CNS) infections.

Use of CI CFZ for perioperative prophylaxis has been evaluated and compared to II for various surgery types. A prospective, randomized study among 20 cardiac surgery patients evaluated a CFZ 2 g IV loading dose (LD) followed by either intermittent administration (n = 10) or 18 h CI perioperatively (n = 10). Mean total CFZ serum concentrations were slightly higher in the CI group compared to the II group at 14.5 h (51.3 ± 18.1 mg/L vs. 34.1 ± 19.2 mg/L, *p* < 0.05) and 24 h (52.5 ± 19.4 mg/L vs. 14.9 ± 10.3 mg/L, *p* < 0.01) post-LD. Mean total myocardial tissue CFZ concentrations were also higher for the CI group (6.9 ± 1.1 mg/L vs. 3.28 ± 0.1 mg/L, *p* < 0.05). More patients achieved fT > MIC for *Escherichia coli* in the CI group (90% vs. 30%, *p* < 0.01) [64]. A controlled trial of 18 patients provided CI CFZ for bariatric surgery and found that body mass index impacted mean adipose tissue CFZ concentrations, with no observations of surgical site infections [65]. A retrospective quasi-experimental cohort study among 516 patients receiving II (n = 284) or CI (n = 232) of CFZ for coronary artery bypass graft surgery found a 66% reduction in SSI with CI compared to II, although it did not reach statistical significance (1.7% vs. 4.6%, *p* = 0.116). There were no differences in safety outcomes between groups [66].

Serum and interstitial CFZ concentrations were compared in seven patients receiving home CI CFZ for uncomplicated cellulitis. The usual starting dose was CFZ 3 g/24 h IV, with a mean daily dose used of CFZ 3.5 g/day IV (36 mg/kg/day). Plasma and interstitial free drug concentrations were not significantly different (17.5 mg/dL vs. 26.6 mg/dL [mean ratio = 0.84, 95% CI 0.696–0.998]), and the lowest free drug concentration observed in the interstitial fluid was 2 mg/dL. The authors concluded that the CI dosing used was appropriate as the lowest observed free drug concentration was above the MIC usually observed in *Staphylococcus* and *Streptococcus* spp. [8].

A retrospective cohort study of 100 patients with bone and joint infections evaluated pharmacokinetic and clinical outcomes of CI CFZ. Patients were administered a CFZ 1 or 2 g LD followed by a CI of 60–80 mg/kg of body weight per day, which was administered over 12 h periods. Dosing was adjusted during the study to achieve a target serum steady-state concentration of 40–70 mg/L. The median daily dose observed was 6 g/day with a median treatment duration of 42 days. The median serum concentration on days 2–10 was 63 mg/L and on days 11–21 was 57 mg/L. Of the 100 patients, 47 required dose adjustment based on serum concentration (9 required dose increases and 38 required dose decreases). Bone concentrations were determined for eight patients, with a median CFZ bone concentration of 13.5 mcg/g and a bone to serum concentration ratio of 0.25. Two patients experienced moderate-grade adverse events. There was no observed CFZ resistance (median follow-up time was 25 months). The authors concluded CI cefazolin would be an ideal agent for prolonged and home therapy due to its effectiveness, safety, convenience, tolerance, and low likelihood for resistance development [9].

Historically, antistaphylococcal penicillins have been recommended over CFZ for the treatment of methicillin-susceptible *Staphylococcus aureus* infection in the CNS; however, there is an increasing body of literature to support the use of optimally dosed CFZ as a safe and effective alternative for a variety of CNS infections [88]. A small retrospective cohort study examined CFZ efficacy for acute bacterial meningitis due to methicillin-susceptible *Staphylococcus aureus* confirmed by cultures or polymerase chain reaction between 2009 and 2019. Seventeen patients received either CFZ or cloxacillin, and cerebrospinal fluid (CSF) drug concentrations were measured. In the CFZ group, eight patients were treated with CI with a median daily dose of 8 g (range 6 to 12 g), and the median CSF concentration for CFZ was 2.8 mg/L. This confirms a therapeutic concentration for CFZ within the CNS, and no therapeutic failures were identified in the CFZ group. The authors concluded that CFZ demonstrated higher-than-expected concentrations in the CNS and achieved therapeutic concentrations adequate for successful treatment of staphylococcal meningitis [89].

#### 3.1.2. Cefuroxime

Cefuroxime (CXM) is a second-generation cephalosporin antibiotic most frequently used for community-acquired upper and lower respiratory tract infections (LRTIs) and, less commonly, for UTIs, ABSSSIs, Lyme disease, and surgical prophylaxis [90]. CXM is excreted unchanged, almost exclusively by the kidneys, with a protein binding of 33–50% [67,91]. The current treatment recommendation for most infections is CXM 1.5 g IV every 8–12 h [90]. However, in critically ill patients, pathogens with high MICs, augmented renal clearance or increased volume of distribution, the traditional dosing regimen may not reach PTA with conventional dosing [17,68,69,91].

An assessment of concept pharmacokinetic evaluation for CI CXM based on plasma, tissue, and bone concentrations was conducted in swine models. The animals received either traditional dosing (CXM 1.5 g IV over 15 min q8h) or CI (CXM 500 mg IV LD over 5 min followed by 1 g IV over the remaining interval time). CI tissue concentrations were consistently lower in the CI group, raising the concern that CI administration of CXM may result in inadequate penetration at the site of infection. While tissue concentrations were consistently lower, plasma concentrations were optimized with the CI (up to 4–5 times the MIC). There was a significantly longer t > MIC in the CI group. In contrast, for higher MICs, short-term infusion had a higher fT > MIC in solid tissues, so the location of the infection may play a key role in CXM administration [69].

In a prospective pharmacokinetic study of CXM in 20 critically ill patients, traditional dosing (CXM 1.5 g IV q8h) was provided and population pharmacokinetic analysis and Monte Carlo dosing simulations were applied with non-linear mixed-effects modeling to evaluate extended infusion (EI) (CXM 1.5 g IV q6-8h over half of dosing interval) and CI (CXM 750 mg IV dosing dose followed by CXM 4500 mg–9000 mg/24 h IV). Traditional dosing demonstrated inadequate target attainment (serum concentrations) in patients with an estimated creatinine clearance ≥ 50 mL/min, with the probability of reaching target attainment decreasing with increasing creatinine clearance. The CI dosing strategy demonstrated that higher-than-normal doses of CXM (up to 9 g/day) over 24 h following a LD is more likely to achieve appropriate targets in patients with preserved or heightened renal function [17].

A prospective, noncomparative trial was conducted in 54 patients receiving CXM for postsurgical prophylaxis following coronary artery bypass grafting procedures. Patients were given a CXM 1.5 g IV LD 30 min prior to surgery followed by a CI of CXM 3 g/day IV until central catheters were removed. All but one (rash and hypotension) of the 54 patients tolerated the CI of CXM. The mean CI serum concentrations in the CI group was 21.6 ± 14.2 mcg/mL, and no patients developed a sternal wound infection. Patients in this study received less of the drug than the amount they would have otherwise received using a traditional dosing model (CXM 8.4 g vs. 12.5 g). Although one patient experienced an adverse event leading to drug discontinuation, CI has previously been shown to result in less infusion-related reactions when compared to traditional dosing (11% vs. 19%) [92].

#### 3.1.3. Ceftriaxone

Ceftriaxone (CRO) is a third-generation cephalosporin that is widely used in the management of infections including community-acquired pneumonia (CAP), pyelonephritis, ABSSSIs, and additional deep-seated infections. Despite its high-volume use, susceptibilities to *Streptococcus pneumoniae*, and common gram-negative pathogens such as *E. coli* have remained stable and high [93]. Furthermore, the extended half-life of up to 8.7 h allows for the recommended once-daily dosing in most infections and populations, including critically ill patients [94,95,96,97] However, there is concern for achieving and maintaining adequate pharmacodynamic targets using once-daily dosing in critically ill patients, especially those with low serum albumin, due to increased renal clearance and volume of distribution in this highly protein-bound drug (83–96%) [95,96]. Additionally, treatment failures have been reported as higher among patients receiving CRO in those with low compared to normal serum albumins (12.3% vs. 7.7%) [94,98].

Among 35 neutropenic cancer patients in a pharmacokinetic study investigating varying dosing strategies, 9 patients received CI ceftriaxone. Patients received a 1 g IV LD over 30 min, followed by 2 g IV every 8 h as a CI (6 g/day). Serum concentrations taken on days 2 through 8 averaged 135 mcg/mL and ranged between 117 and 151 mcg/mL, which far exceeded targets due to the relatively high daily dose used in this study [23].

In a subsequent pilot, clinical outcomes of CI ceftriaxone were evaluated in an intensive care unit (ICU) population. A CI dosing strategy (CRO 2 g/24 h IV) was compared to a traditional dosing strategy (CRO 2 g IV administered as a once daily bolus). Fifty-seven patients were included and there was no difference in clinical cure, bacteriological response, and bacteriological cure between groups. In a subgroup analysis evaluating outcomes for patients who received at least four days of therapy, there was improved clinical curing in patients receiving CI and in patients with lower APACHE-II scores. To our knowledge, no follow-up randomized studies have been conducted to validate these results [24]. A population kinetics study confirmed the previous study’s findings that a 2 g/24 h dose regimen was more likely to achieve adequate serum concentrations when compared with both 2 g IV every 24 h and 2 g IV every 12 h [99].

#### 3.1.4. Ceftazidime

Ceftazidime (CAZ), a third-generation cephalosporin, with broad gram-negative activity but limited activity against gram-positive organisms, is often used in hospital-acquired infections as an anti-Pseudomonal beta-lactam [3]. The recommended dosage is CAZ 1 g IV every 8 h for mild or moderate infection or CAZ 2 g IV every 8 h for severe infections [3].

CI CAZ was compared to intermittent administration in a prospective, randomized, crossover study in critically ill patients with suspected gram-negative infections. Patients were given a CI (CAZ 2 g IV LD followed by CAZ 3 g/24 h IV) or II (CAZ 2 g IV every 8 h) for two consecutive days. After two days, patients from both groups were crossed over and received the opposite regimen. The fT > MIC for the CI was greater than that for bolus dosing (100% and 92%, respectively). However, the area under the bactericidal titer–time curve (AUBC) was less with CI than with bolus dosing [70].

The bactericidal activity of CAZ was investigated in an open-label, randomized, steady-state, four-way crossover study in 12 healthy volunteers. Patients received four unique dosing regimens, two of which were a CI (2 g/24 h or 3 g/24 h). The CI dosing regimens displayed a 100% fT > MIC for *E. coli* and *P. aeruginosa* [71]. Synergistic activity with amikacin has also been confirmed with CI CAZ [100].

Dosing of CI CAZ 100 mg/kg/24h in patients with cystic fibrosis has demonstrated higher PTA and has not appeared to result in sustained resistance [72,73]. Using Monte Carlo simulation, Bulitta et al. confirmed that patients with cystic fibrosis receiving 6 gm/24 h (per 70 kg body weight) would achieve significant PTA to very high MICs up to 12 mcg/mL [29]. Use of CI combined with TDM may also allow for 50% less drug per day in some patients [75]. Additionally, CI for home use delivered via a portable pump has demonstrated good clinical outcomes with a favorable safety profile in patients with cystic fibrosis [73,74].

In a small randomized controlled trial in critically ill patients, CI CAZ given as 12 mg/kg bolus followed by 6 g/24 h resulted in higher sustained targets (concentrations >40 mg/L) compared to traditional II dosing [30]. Some investigators have recommended a CI CAZ dosing strategy of a 2 g LD followed by 3g/24h in patients receiving continuous venovenous hemodiafiltration [101]. 

#### 3.1.5. Ceftazidime/Avibactam

Ceftazidime/avibactam (CAZ/AVI) is a cephalosporin and serum beta-lactamase combination which offers expanded activity against gram-negative organisms and multi-drug resistant (MDR) organisms, including carbapenemase-producing bacteria and metallo-beta-lactamase (MBL)-producing bacteria, in combination with aztreonam. Avibactam is a diazabicyclooctanone (DBO) beta-lactamase inhibitor with unique and reliable activity against *Klebsiella pneumoniae* carbapenemase (KPC)-producing organisms. DBOs demonstrate a linear enzymatic pathway with time-dependent kinetics (%T > threshold concentration to restore beta-lactam activity) [102]. CAZ/AVI was originally approved at a dose of 2.5 g IV every 8 h infused over 2 h for intra-abdominal infections (IAIs) (in combination with metronidazole) and complicated UTIs and has since been approved for hospital- or ventilator-associated pneumonia (HAP/VAP) [102].

A hollow-fiber infection model evaluated 16 unique dosing strategies of the combination of CAZ/AVI plus aztreonam against MBL-producing strains of *E. coli* and *K. pneumoniae*. Of the 16 unique dosing strategies, 6 included a CI of CAZ/AVI 7.5 g/24 h IV. The study was designed to evaluate staggered vs. simultaneous administration of CAZ/AVI plus aztreonam, infusion duration, and aztreonam daily dose on bacterial killing. Continuous infusion and EI of CAZ/AVI also demonstrated higher bacterial killing relative to standard infusion [34].

A retrospective case series was performed to evaluate CI administration of CAZ/AVI among 10 patients with MDR *P. aeruginosa* (n = 6) and *K. pneumoniae* (n = 4) infections of various types. Patients were administered a LD of CAZ/AVI 2.5 g IV followed by CAZ/AVI 5 g/12 h IV q12 h, which could be modified based on patient-specific TDM. The median CAZ plasma concentration was 63.6 mg/L (range 47.6–80 mg/L). All patients met target attainment of at least 4×MIC in plasma. Clinical cur occurred in 80% of patients, and the microbiological eradication rate was 90% [35].

#### 3.1.6. Cefotaxime

Cefotaxime (CTX), a third-generation cephalosporin, is 30–50% protein bound, and unlike other cephalosporins, it has an active metabolite [3,103]. The active metabolite has a longer half-life than that of the parent compound, allowing for an extended dosing interval [3]. It is typically used to treat infections such as UTIs, chronic bronchitis, gram-negative bacteremia, and community and nosocomial LRTIs. The recommended dosage is CTX 3–6 g divided into three daily doses for moderate to severe infections [103].

A randomized, controlled, non-blinded study among 39 patients compared CI (CTX 1 g IV LD followed by CTX 2 g/24) to II (CTX 1 g IV three times daily). The clinical cure rate in both groups was 93% (37/40 and 40/43 in the continuous and intermittent groups, respectively). Time with antibiotic concentrations ≥5× MIC was 100% in the CI group and 55% in the intermittent group (*p* < 0.001) [44]. These results are consistent with other studies showing higher AUC exposure and PTA with CI [44,45,46].

In addition to favorable PK and outcomes, CI CTX has been shown to be financially advantageous compared with traditional dosing with similar clinical and microbiological efficacy. Hitt et al. conducted a cost analysis comparing CI CTX 2 g per day to intermittent daily doses of CRO 1 g daily and found that CI CTX was significantly less costly than intermittent CRO [104].

#### 3.1.7. Cefepime

Cefepime (FEP) is a fourth-generation cephalosporin antibiotic with broad activity including against *P. aeruginosa* and is commonly used for empirical treatment of serious infections such as bacteremia, HAP/VAP, IAIs, and febrile neutropenia [1,105]. FEP is largely renally excreted with a half-life of approximately 2 h and limited protein binding (approximately 16%) [105,106,107]. The recommended dosage for treating serious infections or empirical treatment of critically ill patients is 2 g IV every 8 h over 30 min [2]. One concern with cefepime use is cefepime-related neurotoxicity. Although trough and steady-state concentration thresholds associated with neurotoxicity are not well defined, the literature suggests increasing FEP plasmas concentrations are independently associated with neurotoxicity [46,76,77,108]. Use of CI may provide an opportunity to optimize FEP dosing to achieve an appropriate efficacy threshold while minimizing supratherapeutic exposures that have been associated with FEP-related neurotoxicity [108].

Administration of FEP via CI has demonstrated greater PTA and decreased drug exposure [47,48,76,77,78]. A simulation pharmacodynamic study of 10,000 patients compared intermittent infusion and CI of FEP and piperacillin/tazobactam (PIP/TZB) against extended-spectrum beta-lactamase (ESBL)-producing organisms. The CI dosing regimens (3 g/24 h and 4 g/24 h) enhanced the PTA (60% fT > MIC) compared to the intermittent regimens of FEP 2 g IV every 8 h or every 12 h [78]. An open-label, non-randomized, prospective, observational and descriptive study in which 12 unique dosing strategies were applied via Monte Carlo simulation to date from 15 adult patients with hematological malignancies treated for febrile neutropenia demonstrated similar trends. FEP 6 g/24 h IV demonstrated the highest MIC value for target attainment, which was not improved with increasing daily dose to FEP 8 g IV via 24 h infusion [48]. In contrast, Monte Carlo simulations applied to 266 critically ill adult and pediatric patients found that CI dosing strategies were most likely to achieve targets of fT > 4×MIC, with the FEP 8 g/24 h strategy being the only dosing regimen to achieve >90% probability of target attainment, assuming MIC = 1 mg/L [47].

Cefepime has also been evaluated in combination with several novel beta-lactamase inhibitors such as taniborbactam, enmetazobactam, and xeruborbactam. These broad-spectrum beta-lactamase inhibitors restore the activity of cefepime against a wide range of beta-lactamases, including activity against metallo beta-lactamases for taniborbactam and xeruborbactam. At the time of this review, there is a paucity of data on CI for these combinations [109,110,111].

#### 3.1.8. Ceftaroline

Ceftaroline fosamil (CPT) is a fifth-generation cephalosporin with broad activity including against gram-negative and resistant gram-positive organisms. While ceftaroline is FDA-approved for ABSSSIs and CAP, one of its primary uses in practice is for refractory MRSA infections. Ceftaroline dosing is typically 600 mg every 8–12 h, depending on infection type. It is primarily renally excreted with an average elimination half-life of approximately 2.7 h and has relatively low protein binding (20%) [112]. No dosage adjustment appears to be necessary in obesity [49].

Administration of CPT via CI has limited clinical data; however, a recent observational TDM study evaluated 12 patients who received CPT for confirmed gram-positive infection with various dosing strategies including EI and CI. Among six patients who achieved CI, each achieved a target attainment of 100% fT > 4×MIC. Despite this study’s small sample size, CI CPT demonstrated optimal target attainment and may be considered for use in selected patients [50].

#### 3.1.9. Ceftobiprole

Ceftobiprole (BPR) is a fifth-generation cephalosporin with broad-spectrum activity against gram-positive organisms including MRSA and gram-negative coverage including Enterobacterales and *P. aeruginosa*. Ceftobiprole was FDA approved in 2024 for the treatment of *Staphylococcus aureus* bacteremia, CAP, and ABSSSI [113].

For MRSA bacteremia, BPR is dosed at 500 mg every 6 h for 8 days, followed by 500 mg every 8 h thereafter. The mean half-life of ceftobiprole is approximately 3 h, with minimal protein binding at 16%, and is largely renally excreted with approximately 83% of the active drug recovered in the urine [113].

Clinical data describing the use of ceftobiprole as a CI are lacking; however, a recent pharmacokinetic analysis was conducted in 132 patients to assess the PTA amongst various dosing regimens including EI (over 2 h) and CIs, using manufacturer-recommended standard doses converted into CIs. The authors concluded that for infections caused by MRSA, patients with impaired renal function and augmented renal clearance may benefit from CI BPR to optimize the likelihood of target attainment [54].

#### 3.1.10. Ceftolozane/Tazobactam

Ceftolozane/tazobactam (C/T) is a combination antimicrobial that contains a fifth-generation cephalosporin and a penicillanic acid sulfone beta-lactamase inhibitor. C/T has some gram-positive activity but is primarily marketed for its activity against multidrug-resistant gram-negative pathogens, including *P. aeruginosa*. Although it was initially approved for the treatment of complicated IAIs (in combination with metronidazole) and complicated UTIUTIs, this antimicrobial was recently approved for HAP/VAP [114]. As with most cephalosporins, ceftolozane has a relatively short half-life (2.5–3 h) and limited protein binding of approximately 20% [114].

Approved dosing for C/T ranges from 1.5 to 3 g every 8 h over a 1 h infusion for normal renal function. Evidence to support the safety and efficacy of CI C/T has been described recently. In the available literature, CI dosing ranged from 2.25 g to 9 g/day of C/T [58,59,79,80,81,82,83,84,85]. A prospective cohort study in 72 patients with *P. aeruginosa* infections found that intermittent dosing was inadequate when MICs were ≥4 mg/L, but EI and CI of C/T (dosed 6 g/day) achieved >90% PTA [82]. Five studies incorporated TDM and all demonstrated ceftolozane and tazobactam concentrations that remained above the CLSI breakpoints of 4 mg/L and 0.5 mg/L, respectively [58,83]. Furthermore, drug concentrations also exceeded 4–5x the MIC for 100% of the dosing interval in four of the five studies that evaluated this parameter [58,79,80,83]. All but one encounter in the reported case series and case reports documented clinical resolution with CI C/T [58,59,79,80,81,82,84]. Winans demonstrated CSF concentrations of 83% of serum in a patient with *P. aeruginosa* meningitis receiving a 3 g IV LD followed by 9 g/24 h [85].

#### 3.1.11. Cefiderocol

Cefiderocol (FDC) is a novel siderophore cephalosporin with expanded gram-negative activity to include MDR isolates of Enterobacterales, *P. aeruginosa*, *A. baumannii*, *S. maltophilia*, and other difficult-to-treat gram-negative pathogens including both ESBL-producing and carbapenem-resistant isolates [115]. The terminal half-life is approximately 2.5 h, and the protein binding rate is 58% [115].

Approved dosing for FDC is 2 g IV q8h over a 3 h infusion. No clinical data utilizing CI FDC is available at the time of this review, likely due to its current manufacturer-reported stability of 6 h in normal saline (NS) or 5% dextrose in sterile water (D5W). However, Loeuille and colleagues recently evaluated the physiochemical stability of cefiderocol in polypropylene syringes and found that cefiderocol diluted to 62.5 mg/mL (3 g in 48 mL) in NS or D5W was stable for 12 h at room temperature, retaining >90% of the initial concentration with no visual changes detected. This supports that cefiderocol CI may be feasible to investigate [116].

### 3.2. Cephamycins

#### Cefoxitin

Cefoxitin (FOX) is a parenteral cephamycin antibiotic with gram-positive and gram-negative aerobic and anaerobic activity, which is commonly used in the management of genitourinary and IAI as well as surgical prophylaxis in colorectal procedures [3]. FOX is also recommended as an agent used in combination therapy for many rapid growing nontuberculous mycobacteria (NTM) [117]. FOX is predominantly (80%) renally excreted and has a short half-life of approximately 1 h and a relatively low protein-binding capacity of approximately 35% [3,118]. FOX is commonly dosed at 1–2 g IV every 4 or 6 h over 30 min (maximum 12 g/day), depending on the targeted pathogen and infection [3,117]. Pathogens with high MICs (>16 mcg/mL) and patients with an increased volume of distribution or augmented renal clearance may be subject to suboptimal target attainment with conventional dosing [63,119].

Administration of CI FOX has been associated with several potential benefits compared to traditional dosing. A study conducted in murine models with peritonitis found that CI FOX significantly reduced pro-inflammatory cytokines, TNF-alpha, interleukin, and neutrophil count in the lungs as well as decreased bacterial burden in the serum when compared to intermittent dosing [62]. A retrospective, matched cohort pilot study in 126 patients undergoing colorectal surgery found that rates of surgical-site infections at 30-days post-operatively were numerically lower in patients who received CI FOX when compared to intermittent dosing. Patients were given traditional weight-based FOX IV every 8 h or cefoxitin IV as a CI. The infusion was initiated at the time of surgery, and if continuous, it was given at 3 g over 20 h if <80 kg or 6 g over 20 h if >80 kg. Discontinuation of therapy by 24 h post-operatively, as recommended by national guidelines, was achieved in 100% vs. 84% of patients in the CI and traditional dosing arms, respectively [120].

A case series described three patients with *Mycobacterium abscessus* pulmonary infection who were administered FOX 2 g IV continuously over 8 h with no LD [63]. Only one of the three patients maintained a serum concentration of ≥16 mcg/mL, the susceptibility breakpoint for *M. abscessus*. Use of CI FOX for an active infection warrants further studies, particularly to investigate the use of higher doses (>6 g/day) to achieve target attainment, especially in NTM infections [63].

## 4. Discussion

CI cephalosporins may offer numerous potential clinical and logistical benefits in patients with both gram-positive and gram-negative infections. Although the administration of beta-lactams via CI has yet to show a mortality benefit in an RCT, there have been some limited findings that indicate benefits in relation to clinical cure and no increased adverse events in critically ill patients [6,121,122]. Dulhunty et al. conducted an open-label, international RCT which included 7031 critically ill adult patients with sepsis and randomized patients to receive either continuous (n = 3498) or intermittent (n = 3533) infusion of either piperacillin–tazobactam or meropenem. The primary outcome of 90-day all-cause mortality occurred in 24.9% of patients in the CI group and 26.8% of patients assigned to the II group (odds ratio 0.91 [95% CI 0.81–1.01]; *p* = 0.08). Achieving a clinical cure at 14 days after randomization occurred at a significantly higher rate for the CI group at 55.7% as compared to a rate of 50.0% in the II group (absolute difference 5.7% [95% CI 2.4% to 9.1%]) [6]. Although cephalosporins were not included in this study, these data highlight the potential benefit of CI beta-lactams with no increase in the incidence of adverse events. Clinicians should consider the use of CI cephalosporins when clinically appropriate. 

In addition to their clinical implications, CI cephalosporins offer several other benefits. They reduce the frequency of IV site manipulation as well as the nursing time required for administrations, thus with presumed potential for risk reduction of catheter-related bloodstream infections. Moreover, they enable the use of a portable pump in the outpatient setting, enhancing patient mobility which can be an attractive option for patients receiving outpatient parenteral antimicrobial therapy. Given their time-dependent bactericidal activity, CIs optimize the PTA of beta-lactams even in the presence of inter- and intra-patient pharmacokinetic variability [2,3]. While there may be concerns about an increased number of adverse events associated with prolonged infusion due to higher serum and tissue drug concentrations, this has not been substantiated by available data [4,5,6,121,122]. Furthermore, these elevated concentrations may facilitate the utilization of lower total daily doses of cephalosporins, resulting in potential cost savings [28,75]. Our CI dosing recommendations, based on the available data for each cephalosporin are presented in Table 1.

## 5. Conclusions

The available data describing the PK, outcomes, and logistics of delivering cephalosporins via CI support the opportunity to optimize cephalosporin delivery. These data may be leveraged for both inpatient and outpatient use, as well as by local antimicrobial stewardship teams, informing dosing strategies to optimize drug exposure and possibly aid in cost minimization and improve patient placement and satisfaction. Further research in this area may offer more insight as to when CI should be prioritized, especially as the clinical benefit remains unclear.

## Figures and Tables

**Table 1:** Continuous infusion cephalosporins dosing recommendations.

Agent	Dosing Recommendation for CI	Storage/Stability	Notes/Special Populations *	MIC_90_ of Relevant Pathogens (mcg/mL)
Cefazolin (CFZ) [7,8,9]	CFZ 2 g IV LD followed by CI of 60–80 mg/kg/day Maximum daily dose: 12 g	Reconstituted solutions are stable for 1 day at room temperature and for 10 days under refrigeration. Protect powders and reconstituted solutions from light. Parenteral admixtures are stable for 48 h at room temperature and 14 days when refrigerated.	Doses may be adjusted for body weight. Consider therapeutic drug monitoring to target serum concentrations of 40–70 mg/L.	MSSA: <2 *E. coli*: 1.6 *K. pneumoniae*: 4 *Enterobacter* spp.: >32 *H. influenzae*: 16 *Streptococcus* spp.: ≤2
Cefuroxime (CXM) [10,11,12,13,14,15,16,17]	CXM 1.5 g IV LD followed by CI of 3 g/24 h Maximum daily dose: 4.5 g	Reconstituted solution with NS or D5W is stable for 24 h at room temperature, 7 days when refrigerated, or 26 weeks when frozen. Store intact vials at 15–30 °C (59–86 °F) and protect them from light.	Dose adjustments should be considered in patients with renal impairment.	MSSA: 2 *E. coli*: 8 *K. pneumoniae*: >16 *Enterobacter* spp.: >16 *H. influenzae*: 4 *S. pneumoniae*: 8 Viridans streptococci: 4 Beta-hemolytic streptococci: 0.25
Ceftriaxone (CRO) [18,19,20,21,22,23,24]	CRO 500 mg IV LD followed by 2 g/24 h Maximum daily dose: 6 g	Thawed premixed solutions (manufacturer premixed) are stable for 3 days at 25 °C (77 °F) or for 21 days at 5 °C (41 °F). Reconstituted solution (100 mg/mL) with NS, D5W, or SWFI is stable for 2 days at room temperature or 10 days when refrigerated. Prior to reconstitution, store powder for injection at ≤25 °C (≤77 °F) and protect from light.		MSSA: 4 *E. coli*: 4–8 *K. pneumoniae*: >8 *S. pneumoniae*: 1 Viridans streptococci: 1 Beta-hemolytic streptococci: 0.06–0.12 *N. meningitidis*: <0.0002 *H. influenzae*: ≤0.06 *M. catarrhalis*: 0.5 *P. mirabilis*: ≤0.25
Ceftazidime (CAZ) [25,26,27,28,29,30]	CAZ 2 g IV LD followed by 6 g/24 h Maximum daily dose: 12 g	Thawed solution in NS in a Viaflex is stable for 24 h at room temperature and for 7 days after refrigeration. If reconstituted in NS D5W, D5NS, LR, or D10W, it is stable for 24 h at room temperature and for 7 days when refrigerated. Reconstituted and further diluted solutions are stable for 24 weeks when frozen at −20 °C (−4 °F). Vials should be stored at 20–25 °C (68–77 °F) and protected from light.		*E. coli*: ≤2 *Enterobacter* spp.: >16 *Klebsiella* spp.: ≤2 *H. influenzae*: ≤0.25 *M. catarrhalis*: 0.5 *P. mirabilis*: ≤0.12 *P. aeruginosa*: 16 *Serratia* spp.: 0.25 *S. maltophilia*: >16
Ceftazidime/avibactam (CAZ/AVI) [18,31,32,33,34,35]	CAZ/AVI 2.5 g IV LD followed by CAZ/AVI 10 g/24 h Maximum daily dose: 15 g	Reconstitute vial with 10 mL of NS, D5W, SWFI, or other compatible solution. Mix and further dilute to a concentration of 8–40 mg/mL CAZ and 2–10 mg/mL AVI. Store intact vials at 25 °C. After reconstitution, transfer to infusion bag within 30 min for further dilution. Admixed solutions (up to dextrose 2.5% and sodium chloride 0.45%) are stable up to 12 h at room temperature and 24 h at 2 °C to 8 °C.		*Citrobacter* spp.: 0.12 *Enterobacter* spp.: 0.5 *E. coli*: 0.12 *E. coli* (ESBL phenotype): 0.25 *H. influenzae*: 0.03 *K. pneumoniae*: 0.5 *K. pneumoniae* (ESBL phenotype): 1 *K. pneumoniae* (meropenem NS): 2 *M. catarrhalis*: 0.12 *P. mirabilis*: 0.06 *P. aeruginosa*: 4 *P. aeruginosa* (meropenem NS): 16 *P. aeruginosa* (XDR): 32
Cefotaxime (CTX) [11,25,36,37,38,39,40,41,42,43,44,45,46]	CTX 1 g IV LD followed by 2–4 g/24 h Maximum daily dose: 8 g	Reconstituted solution stable for 12–24 h at room temperature, 7–10 days when refrigerated, and 13 weeks when frozen. IV infusions in NS or D5W solution are stable for 24 h at room temperature, 5 days when refrigerated, or 13 weeks when frozen in Viaflex plastic containers. Thawed solutions of frozen mixed bags are stable for 24 h at room temperature or 10 days when refrigerated. Store vials in temperatures below 30 °C (86°) and protect from light.	A dosing range of 0.5 to 2 g in 12 h intervals may be suitable for non-immunocompromised patients without CNS infections.	MSSA: 4 *S. pneumoniae*: 1 Viridans streptococci: 1 Beta-hemolytic streptococci: ≤0.06 *Citrobacter* spp.: 128 *Enterobacter* spp.: 256 *E. coli*: ≤1 *K. pneumoniae*: ≤1 *N. meningitidis*: 0.007 *H. influenzae*: ≤0.015 *M. catarrhalis*: 1 *P. mirabilis*: ≤1 *Serratia* spp.: 128
Cefepime (FEP) [25,27,42,43,47,48]	FEP 2 g IV LD followed by 4–6 g/24 h Maximum daily dose: 6 g (note, 8 g has been used in patients with augmented clearance)	After reconstitution with NS or D5W, it is stable for 24 h at room temperature or 7 days when refrigerated. Intact vials must be stored at 20–25 °C (68–77 °F) and protected from light.	Dose adjustments should be considered in patients with renal impairment and those with augmented renal clearance (potentially up to 8 g).	MSSA: 4 *S. pneumoniae*: 1 Beta-hemolytic streptococci: ≤0.12 Viridans streptococci: ≤0.12 *Citrobacter* spp.: ≤0.25 *Enterobacter* spp: ≤1 *E. coli*: ≤0.25 *H. influenzae*: ≤0.25 *Klebsiella* spp.: ≤0.25 *M. morganii*: ≤0.25 *P. mirabilis*: ≤1 *P. aeruginosa*: 16 *Serratia* spp.: ≤0.25
Ceftaroline (CPT) [12,18,19,21,49,50]	CPT 600 mg IV LD followed by 1.2 g/24 h Maximum daily dose: 1.8 g	After reconstitution in 1/2NS, D5W, LR, or NS, it must be used within 6 h when stored at room temperature or within 24 h if refrigerated at 2–8 °C (36–46 °F). Vials must be stored at 25 °C (77 °F).	Dose adjustments should be considered in patients with renal impairment.	MSSA: 0.25 MRSA: 1 *S. pneumoniae*: 0.12 Viridans streptococci: 0.12 Beta-hemolytic Streptococci: ≤0.015 *E. coli*: 0.25 *K. pneumoniae*: 8 *P. mirabilis*: 0.25 *Serratia* spp.: 2
Ceftobiprole (BPR) [20,51,52,53,54]	BPR 500 mg LD followed by 2 g/24 h Maximum daily dose: 3 g	After reconstitution in NS, may store for 4 h at 25 °C (77 °F) or 24 h at 2 °C to 8 °C (36–46 °F). After reconstitution in D5W, may store for 6 h at 25 °C (77 °F) or 94 h at 2 °C to 8 °C (36–46 °F). Reconstituted solution should be protected from light. Vials must be stored at 2–8 °C (36–46 °F) and protected from light. Reconstituted solutions may store for ≤1 h at room temperature and ≤24 h refrigerated prior to further dilution in an infusion bag.	Dose adjustments should be considered in patients with renal impairment.	MSSA: 0.5 MRSA: 2 *S. pneumoniae*: ≤0.015 Viridans streptococci 0.25 Beta hemolytic Streptococci ≤ 0.06 *E. coli*: 0.06 *K. pneumoniae* 0.06 *P. mirabilis*: ≤0.06 *P. aeruginosa*: 16 *Serratia* spp.: 8
Ceftolozane/tazobactam (C/T) [55,56,57,58,59]	C/T 3 g IV LD followed by 4.5 g–6 g/24 h Maximum daily dose: 9 g	Diluted solutions can be stored for 24 h at room temperature or for 7 days at 2–8 °C (36–46 °F). Vials must be stored at 2–8 °C (36–46 °F) and protected from light. Reconstituted solutions can be held for 1 h prior to placement and further dilution into an infusion bag.		*S. aureus*: 32 *S. pneumoniae*: 0.125–16 *Citrobacter* spp.: 8 *Enterobacter* spp.: 8 *E. coli*: 0.5 *E. coli* (ESBL phenotype): 4 *K. pneumoniae*: >32 *K. pneumoniae* (ESBL phenotype): >32 *P. mirabilis*: 0.5 *P. aeruginosa*: >32 *P. aeruginosa* (MDRS): >32 *P. aeruginosa* (XDR): >32 *Serratia* spp.: 1
Cefoxitin (FOX) [7,60,61,62,63]	FOX 2 g IV LD followed by either 3 g/24 h (if ≤80 kg) or 6 g/24 h (if >80 kg) Maximum daily dose: 8 g	Prior to reconstitution, store at 2–25 °C (36–77 °F). Reconstituted solution in SWFI, BWFI, NS, or D5W is stable for 6 h at room temperature or for 7 days when refrigerated.	≥6 g/day is likely required for most rapidly growing mycobacterial organisms, especially in deep-seated infections.	MSSA: 4 *Enterobacter* spp.: 256 *E. coli*: 8 *K. pneumoniae*: 16 *H. influenzae*: 4 *M. morganii*: 32 *P. mirabilis*: 4 *Serratia* spp.: 64

* A pharmacist should evaluate the safety of compatibility and determine if there is considerable advantage to mixing agents or administering concomitant IV medications through the same line. LD: loading dose; h: hour; CNS: central nervous system; NS: normal saline; D5W: dextrose 5% in water; SWFI: sterile water for injection; BWFI: bacteriostatic water for injection; CI: continuous infusion; ESBL: Extended-spectrum beta-lactamase; NS: non-susceptible.

**Table 2:** Selection of Published in vivo and in vitro evidence of CI cephalosporins.

Study	Type of Study	Population	Comparator Arms/Groups	PK/PD Data and Outcomes
*Cefazolin*				
Howard GW, et al. [8]	Observational trial	*n* = 7; patients with uncomplicated cellulitis	No LD provided. CFZ 3 g/24 h IV for ≥5 d, adjusted at the discretion of the physician.	Mean (±SD) dose of CFZ 3.5 ± 1.1 g (36 ± 6.1 mg/kg) IV via CI. Total concentrations (mean ± SD) in plasma proved higher than interstitial fluid concentration in 6/7 patients (32 ± 17 mg/L vs. 17.4 ± 8.3 mg/L). Free drug concentrations were not significantly different between plasma and interstitial fluid. Positive correlation between free concentrations of plasma and interstitial fluid (*p* = 0.005).
Zeller V, et al. [9]	Retrospective cohort study	*n* = 100; patients with bone and joint infection	CFZ 1 or 2 g IV LD (for daily doses ≤ 4 g or >4 g, respectively) followed by CFZ 60–80 mg/kg/24 h IV.	Median CFZ serum concentration 63 mg/L on days 2–10 and 57 mg/L on days 11–21 (target 40–70 mg/L); median CFZ bone concentration of 13.5 µg/g (n = 8). Cure/probable cure in 93% of patients. One person died secondary to infection.
Adembri, et al. [64]	Prospective, randomized study	*n* = 20; cardiac surgery patients	CFZ 2 g IV LD, followed by either CFZ 1 g IV q6h x 3 doses (at 3, 9, and 15 h after the first dose) (n = 10) or CFZ 3 g/18 h IV (n = 10).	Mean total CFZ serum concentrations were significantly higher with CI compared to II at 14.5 h (51.3 ± 18.1 mg/L vs. 34.1 ± 19.2 mg/L, *p* < 0.05) and 24 h post dose (52.5 ± 19.4 mg/L vs. 14.9 ± 10.3 mg/L, *p* < 0.01). Mean total myocardial tissue CFZ concentrations higher for CI group (6.9 ± 1.1 mg/L vs. 3.28 ± 0.1 mg/L, *p* < 0.05). More patients in the CI group achieved free concentrations 90% T > MIC (assuming E. coli) (90% patients vs. 30% patients, *p* < 0.01).
Anlicoara R, et al. [65]	Observational trial	*n* = 18; patients undergoing bariatric surgery	CFZ 2 g IV LD followed by CFZ 1 g IV over 2 h during surgery.	Mean adipose tissue CFZ concentration at start of surgery = 6.66 ± 2.56 mg/L and at surgery conclusion = 7.93 ± 2.54 mg/L; higher initial and final tissue concentrations with BMI < 40 kg/m^2^. No SSIs in BMI ≥ 40 kg/m^2^.
Shoulders BR, et al. [66]	Retrospective quasi-experimental cohort study	*n* = 516; patients undergoing CABG on CPB	CFZ 2 or 3 g IV q2h (n = 284) vs. CFZ 2 or 3 g/24 h IV (n = 232) during cardiac surgery *Initial dosage adjustments for CrCl*.	No statistically significant difference in the reduction in SSI in the CI group vs. II group (1.7% vs. 4.6%, p = 0.116). No statistically significant difference in safety outcomes, such as seizures, AKI, or need for postoperative dialysis, between groups.
*Cefuroxime*				
Broekhuysen et al. [67]	Controlled trial	*n* = 18; patients > 70 years old with acute pulmonary infection	CXM 1500 mg IV LD followed by CXM 4500 mg/24 h IV (n = 7) vs. CXM 4500 mg IV daily divided q8h or q12h (n = 11) (all doses adjusted for CrCl) for an average of 7 days.	Mean (range) C_ss_ in the CI group was 37 mg/mL (23–61 mg/L). Mean (range) C_max_ and C_min_ in the II group were 83 mg/L (44–118 mg/L) and 10 mg/L (1.6–29.5 mg/L), respectively.
Pass et al. [68]	Prospective, non-comparative trial	*n* = 54; patients undergoing CABG procedure	CXM 1500 mg IV 30 min preoperatively followed by 3000 mg/24 h IV (average duration 2.6 ± 2.1 days).	Mean (±SD) C_ss_ 21.6 ± 14.2 mg/L (range 6.56–59.5 mg/L). Significant inverse correlation between estimated CrCl and serum concentration (r = −0.5029; *p* = 0.0005). No patients experienced sternal wound infection within 30 days post-op or readmission for sternal wound infection within 6 months.
Carlier et al. [17]	Observational PK study	*n* = 20; patients in the ICU from which 160 blood samples were collected	CXM 1500 mg IV q8h (750 mg IV q8h for CrCl < 20 mL/min), with population PK analysis and Monte Carlo dosing simulations applied with non-linear mixed-effects modeling to evaluate EI (no LD, CXM 1500 mg q6-8 h over half of the dosing interval) and CI (CXM 750 mg IV LD followed by CXM 4500 mg–9000 mg/24 h IV).	Standard intermittent dosing of CXM resulted in inadequate PTA (87%) for MICs of 8 mg/L in patients with CrCl ≥ 50 mL/min. CrCl ranged 10–304 mL/min. The PTA decreases as CrCl increases; thus, standard II doses may be insufficient in critically ill patients. PTA was overall improved with simulated CI dosing strategies. PTA ≥87% for CI of 9 g daily and CrCl ≤ 200 mL/min.
Tøttrup M, et al. [69]	PK study in swine models	*n* = n/a; plasma, tissue, and bone concentrations were assessed	CXM 1500 mg IV once vs. CXM 500 mg IV LD followed by CXM 1000 mg/8 h IV.	Tissue penetration was incomplete in all groups except subcutaneous tissue penetration in the II group. Plasma concentrations consistently optimized in CI group with longer T > MIC.
*Ceftriaxone*				
Salvador P, et al. [23]	PK study	*n* = 35; patients with neutropenia	High variability in dosing strategies, up to CRO 6 g/24 h IV. Most commonly used was LD 1 g followed by 2 g/8 h (repeated).	High variability in PK observations due to high variability in dosing regimens. Mean serum CRO concentration on day 2–8 was 135 mg/L (range 117–151 mg/L).
Roberts JA, et al. [24]	Open-label, randomized controlled pilot study	*n* = 57; patients in the ICU diagnosed with sepsis	CRO 2 g IV once daily vs. CRO 2 g/24 h IV.	No statistically significant difference in the intention-to-treat analysis for clinical response, clinical cure, or bacteriological response. Controlling for SOFA score and age demonstrated improved clinical outcomes among CI group (aOR 22.8, 95% CI 2.24–232.3, *p* = 0.008) and among those with low APACHE score (aOR 0.70, 95% CI 0.54–0.91, *p* = 0.008).
*Ceftazidime*				
Benko AS, et al. [70]	Prospective, randomized, crossover study	*n* = 14; patients with suspected gram- negative infection (mostly pneumonia)	CAZ 2 g IV LD followed by CAZ 3 g/24 h IV vs. CAZ 2 g IV q8h; participants received each regimen for 2 days prior to crossover to opposite regimen.	Mean serum C_max_ for II was 124.4 ± 52.6 mg/L, mean serum C_min_ was 25.0 ± 17.5 mg/L. Mean C_ss_ for CI was 29.7 ± 17.4 mg/L. Time > MIC was higher in CI group than II (T > MIC 100% vs. 92%).
Nicolau DP, et al. [71]	Open-label, randomized, steady-state, four-way crossover study	*n* = 12; healthy volunteers	CAZ 1 g IV q8h vs. CAZ 1 g IV q12h vs. CAZ 3 g/24 h IV vs. CAZ 2 g/24 h IV.	AUBCs for all organisms were the same for II and CI doses (*p* > 0.05). No statistically significant differences found for varying CAZ dosing schedules for any isolates obtained from blood samples (*p* > 0.05).
Riethmueller J, et al. [72]	Randomized, crossover study	*n* = 80; patients with cystic fibrosis colonized with *P. aeruginosa*	CAZ 200 mg/kg/day in 3 divided doses IV with TOB 10 mg/kg OR CAZ 100 mg/kg/24 h IV with TOB 10 mg/kg via a 30 min IV infusion.	CI mean concentrations 32 ± 12 mg/L (target of >20 mg/L). Mean peak concentrations of II were 159 ± 44 mg/L (target < 180 mg/L) while mean trough concentrations were 8.5 ± 5 mg/L (target < 30 mg/L).
Vinks AA, et al. [73]	Observational cohort study	*n* = 17 patients with cystic fibrosis	CAZ CI 100 mg/kg/24 h IV given via infusion pump at home	25 clinically evaluable courses among 12 patients were all considered effective over a mean duration of 21 days; Bacterial density and proportion of patients with positive cultures decreased significantly; Among 10 patients with TDM, mean serum concentrations were 28.4 ± 5.0 mg/L and sputum concentrations were 3.9 ± 4.0 mg/L
Rappaz I, et al. [74]	Observational cohort study	*n* = 14 pediatric patients with cystic fibrosis and chronic *P. aeruginosa* infections	CAZ CI 100 mg/kg/24 h IV given via infusion pump at home or CAZ standard II	Among 14 children (mean weight 38.8 kg, mean age 12.6 years), CAZ CI maintained mean serum concentrations of 29.7 ± 9.9 mcg/mL and 27.4 ± 6.6 mcg/mL on days 3 and 10., respectively which achieved target concentrations significant more frequently than II. Mean sputum concentrations were 2.1 ± 1.1 mcg/g in patients receiving CI, very similar to those achieved with II. No resistance was noted and CI was well tolerated.
Bosso JA, et al. [75]	Prospective, crossover pilot study	*n* = 5 patients with cystic fibrosis requiring IV therapy for exacerbation	CAZ II 2 g q8 for 10 days and crossed over at next hospitalization to CAZ CI adjusted via TDM to achieve concentrations 6.6 × the MIC of least susceptible isolate	No differences in laboratory values, clinical outcomes or bacterial density; the mean reduction in CAZ dosage needed to obtain target concentrations using the CI was 50%
Bulitta JB, et al. [29]	Pharmacokinetic study with Monte Carlo simulation	*n* = 15; 8 patients with cystic fibrosis and 7 healthy volunteers	Patients received 2 g IV over 5-min infusion; Monte Carlo simulation of multiple dosing strategies including standard II, EI over 5-h and CI of 6 g/24 h	Based on Monte Carlo simulations, standard II dosing (2g q8h) over 30 min achieved good PTA for MICs of ≤ 1 MIC in patients with CF; using EI of 2 g q8h over 5-h, PTA remained high for MICs approaching 12 mg/L; Use of CI 6 g/24 h resulted in high PTA for MICs ≤ 12 mg/L. All simulations assumed 2 g/70 kg.
Lipman J, et al. [30]	Randomized controlled trial	*n* = 18; critically ill patients	CAZ 12 mg/kg LD followed by CAZ 6 g/24 h IV CI vs. CAZ 2g q8h II	Target concentrations were to remain above 40 mg/L in the study; all patients except 1 receiving CI met the goal versus target attainment in only 20–30% of those receiving standard II dosing
El Haj C, et al. [28]	Pharmacokinetic analysis	*n* = n/a; CAZ susceptible and resistant *P. aeruginosa* isolates	CAZ 6 g/24 h OR CAZ 9 g/24 h.	CAZ exhibited dose-dependent antibiofilm activity in vitro; administration of CAZ by CI may provide benefits over intermittent bolus infusion.
*Ceftazidime/Avibactam*				
Goncette V, et al. [35]	Retrospective case series	*n* = 10; MDR *P. aeruginosa* (n = 6) and *K. pneumoniae* (n = 4) (multi-site/source)	CAZ/AVI 2.5 g IV LD followed by CAZ/AVI 5 g/12 h IV given q12h (i.e., 10 g/24 h CI); *Initial dosage adjustments for CrCl and subsequent dosage adjustments based on TDM*.	Median CAZ plasma C_ss_ was 63.6 mg/L (range 47.6–80 mg/L). Moreover, 100% of patients met goal of ≥4× MIC in plasma and/or site infection., and 40% of patients received additional antibiotics. Clinical cure was 80%, and microbiological eradication was 90%. The 30-day mortality was 10% (1 patient death attributed to unrelated cause of ventilator-associated tracheobronchitis).
Lodise TP, et al. [34]	Hollow-fiber infection model	*n* = n/a; MBL-producing strains of *E. coli* and *K. pneumoniae*	Staggered vs. simultaneous administration of CAZ/AVI plus aztreonam; 16 unique dosing strategies, of which 6 included CAZ/AVI CI: CAZ/AVI 7.5 g/24 IV + various aztreonam dosing strategies.	Simultaneous administration was superior to staggered administration against MBL-producing organisms. Longer infusion durations (2 h infusions and CI) demonstrated enhanced bacterial killing compared to standard infusion.
*Cefotaxime*				
Buijk SE, et al. [44]	Non-randomized, block design, observational study	*n* = 15; patients undergoing elective orthotopic liver transplantation	CTX 4 g/24 h IV vs. 1 g IV q6h as an II, aimed to determine the PK of CTX in serum, bile and urine.	Mean concentration in serum after CI was 18 mg/L. Serum concentrations of ≥4 mg/L were achieved for 100% of the CI dosing interval and for 60% of the II interval.
van Zanten, et al. [45]	Randomized controlled, prospective, non-blinded study	*n* = 93; patients with acute exacerbations of chronic obstructive pulmonary disease	CTX 1 g IV LD followed by CTX 2 g/24 h IV vs. CTX 1 g IV three times daily.	Clinical cure did not differ between groups (93%). Time ≥ 5x MIC was 100% in the CI group and 55% in the intermittent group (*p* < 0.001).
Seguin P, et al. [46]	Prospective observational study	*n* = 11; patients in the ICU with secondary peritonitis	CTX 4 g/24 h IV, aimed to determine SS plasma and peritoneal concentration of CTX.	CI of CTX at 4 g/day provided mean plasma and peritoneal concentrations well above MIC for the gram-negative bacteria discovered (24.0 ± 21.5 on day 2 and 22.1 ± 20.7 on day 3).
*Cefepime*				
Burgess DS, et al. [76]	Randomized crossover study	*n* = 12; healthy volunteers	FEP 2 g IV q12h via II vs. FEP 3–4 g/24 IV.	Intermittent infusion regimen achieved serum concentrations above the MIC for *P. aeruginosa* and *E. cloacae* in 11 patients for ≥70% of the dosing interval when MIC was ≤4 mcg/mL. Steady state concentrations for both CI regimens (i.e., 3 and 4 g/24 h) were above the MIC for *P. aeruginosa*, *E. cloacae*, and *S. aureus*, but C_ss_ was ≥4x MIC only if the MIC was ≤2 mcg/mL.
Boselli E, et al. [77]	Prospective, open-label study	*n* = 20; patients with severe VAP	FEP 2 g IV LD followed by FEP 4 g/24 h IV.	Mean plasma C_ss_ was 13.5 ± 3.3 mg/L, and mean epithelial lining fluid C_ss_ was 14.1 ± 2.8 mg/L. Mean percentage penetration to epithelial lining was 100%.
Al-Shaer MH, et al. [47]	Monte Carlo simulation	*n* = 266; critically ill pediatric and adult patients	A total of 8 unique dosing strategies evaluated via Monte Carlo simulation, 3 of which included CI: FEP IV 6 g/24 h, 7 g/24 h, and 8 g/24 h. EIs of FEP 4 g IV via 4 h infusion as LD to CI regimens were also evaluated.	CI dosing strategies were most likely to achieve targets of Time > 4×MIC, with only FEP 8 g/24 h IV achieving >90% PTA (MIC = 1 mg/L). Assuming higher MIC (8 mg/L), the regimen with the highest PTA was LD provided over EI followed by CI.
Alvarez JC, et al. [48]	Open-label, non-randomized, prospective, observational, and descriptive study	*n* = 15; patients with hematological malignancies treated for febrile neutropenia	12 unique dosing strategies evaluated via Monte Carlo simulations, 3 of which included CI: FEP IV 4 g/24 h, 6 g/24 h, and 8 g/24 h.	PTA was higher among CI regimens. FEP 6 g/24 h IV obtained the highest cut-off MIC value.
Reese A, et al. [78]	Monte Carlo simulation	*n* = 10,000; non-duplicate ESBL isolates	Intermittent bolus and CI of PIP/TZB and FEP (1 g q8h, 1 g q12h, 2 g q12h, 3 g/24 h and 4 g/24 h).	FEP 4 g CI had the highest PTA (T > MIC = 77%); no CI regimen achieved an adequate (>90%) T > MIC.
*Ceftaroline*				
Fresán D, et al. [50]	Retrospective, observational study	*n* = 12; patients receiving treatment for confirmed gram-positive infections	1800 mg/24 h, 1200 mg/24 h, and 600 mg/24 h. All doses adjusted based on renal function.	Six of seven patients who received CI achieved 100% fT > 4×MIC. Based on TDM, two patients receiving 1800 mg/24 h via continuous infusion had the dose decreased, while others were maintained.
*Ceftobiprole*				
Cojutti PG, et al. [54]	Retrospective pharmacokinetic study with Monte Carlo simulation	*n* = 132; patients with gram-positive infections	The following CI dosing strategies were evaluated via Monte Carlo simulation: 3000 mg/24 h for eGFR > 130 mL/min/1.73 m^2^; 2000 mg/24 h for eGFR 51–130 mL/min/1.73 m^2^; 1500 mg/24 h for eGFR 30–50 mL/min/1.73 m^2^; 750 mg/24 h CI for eGFR < 30 mL/min/1.73 m^2^.	Monte Carlo simulations of CI, using standard doses based on GFR, were needed to achieve optimal PD targets against MRSA. This remained the case for patients with renal impairment and augmented renal clearance.
*Ceftolozane/Tazobactam*				
Jones BM, et al. [79]	Case report	*n* = 1; MDR *P. aeruginosa* pulmonary infection	C/T 4.5 g/24 h.	Clinical and microbiological resolution; no TDM performed.
Stewart A, et al. [80]	Case report	*n* = 1; MDR *P. aeruginosa* cavitating pulmonary infection	C/T 4.5 g/24 h.	Clinical resolution; improvement of lesion on imaging; inpatient and outpatient TDM demonstrated unbound plasma (and assumed epithelial lining) ceftolozane concentrations well above 4–5 times the MIC value associated with maximal bacterial killing for the full dosing interval.
Davis SE, et al. [81]	Case report	*n* = 1; cystic fibrosis patient with *P. aeruginosa* and ESBL *E. coli*	C/T 3 g IV LD followed by C/T 6 g/24 h.	TDM confirmed adequate exposure: observed concentrations exceeded the established CLSI susceptibility breakpoints for P. aeruginosa and E. coli (≤4/4 μg/mL and ≤2/4 μg/mL, respectively).
Pilmis B, et al. [82]	Prospective cohort study	*n* = 72; patients with MDR *P. aeruginosa* infections (primarily respiratory)	C/T 3 g IV q8h infused over 1 h (n = 44) vs. C/T 3 g IV q8h via 4 h EI (n = 13) vs. C/T 9 g/24 h (n = 15).	No difference in PTA for MICs < 4 mg/L; intermittent dosing inadequate for MICs ≥ 4 mg/L, but prolonged and CI of C/T (dosed 6 g/day) achieved >90% PTA.
Sime FB, et al. [83]	Prospective observational study with Monte Carlo Simulation	*n* = 12; critically ill patients without renal impairment	C/T 1.5 g IV or 3 g IV q8h infused over 1 h (n = 1000 Monte Carlo Simulations).	CI C/T dosing regimens associated with higher PTAs particularly in patients with augmented renal clearance (85% for directed therapy with MICs up to 4 mg/L; 84 and >85%, for empirical coverage with MICs up to 64 mg/L with 1.5 g and 3 g dosing regimens, respectively).
Jones BM, et al. [84]	Retrospective, case series	*n* = 7; Outpatients with *P. aeruginosa* infections (multi-site/source)	C/T 4.5 g/24 h (n = 6) and C/T 9 g/24 h (n = 1) (labeled dosing converted to 24 h dosing, e.g., patients eligible for 1.5 g q8h received 4.5 g CI).	6 of 7 patients had symptom resolution; 3/3 patients had microbiological clearance.
Otero JA, et al. [59]	Case report	*n* = 1; MDR *P. aeruginosa* osteomyelitis	C/T 1.5 g IV LD followed by 2.25 g/24 h (adjusted for renal dysfunction, glomerular filtration rate 40 mL/min).	Clinical resolution (both antibiotic and surgical management employed).
Sheffield M, et al. [58]	Retrospective, case series	*n* = 7; deep-seated MDR *P. aeruginosa* infections	C/T 3 g IV LD for all patients; 1 patient treated with C/T 3 g/24 h (n = 1, suppression dosing); C/T 4.5 g/24 h (n = 1, adjusted for renal function; C/T 6 g/24 h (n = 5, 3 patients had q12h infusions over 12 h).	7/7 patients had clinical resolution, and 1/1 patients had partial microbiological clearance; TDM in 4 cases confirmed adequate exposure with observed concentrations 100% fT > 4×MIC.
Winans SA, et al. [85]	Case report	*n* = 1; *P. aeruginosa* meningitis	C/T 3 g IV LD followed by C/T 9 g/24 h.	Serum TDM obtained 3 and 6.75 h after the 3 g LD of C/T and 15 h after CI started. CSF TDM obtained 4 h after the third serum concentration was obtained and 6 d after starting CI to confirm steady state. Ceftolozane CSF concentrations were 83% of serum. Clinical resolution with C/T + IV and intraventricular gentamicin.
*Cefiderocol*				
No clinical data available				
*Cefoxitin*				
Suffoletta TJ, et al. [76]	Retrospective, cohort-matched pilot study	*n* = 116; patients undergoing colorectal surgery stratified into low and medium risk groups	FOX 1 g IV if ≤80 kg or 2 g IV if >80 kg q8h for three doses 3 h after surgery vs. FOX 3 g/20 h IV if ≤80 kg or 6 g/20 h IV if >80 kg started immediately after surgery.	30-day postoperative SSI rate showed a 50% relative risk reduction in medium-risk patients while it was equal between continuous and intermittent regimens in the low-risk group.

CI: continuous infusion; EI: extended infusion; II: intermittent infusion; SSI: surgical site infection; IV: intravenous; TDM: therapeutic drug monitoring; LD: loading dose; h: hour; PTA: probability of target attainment; CSF: cerebrospinal fluid; MIC: minimum inhibitory concentration; ICU: intensive care unit; ESBL: Extended-spectrum beta-lactamase.
